# Valorization of Side Stream Products from Sea Cage Fattened Bluefin Tuna (*Thunnus thynnus)*: Production and In Vitro Bioactivity Evaluation of Enriched ω-3 Polyunsaturated Fatty Acids

**DOI:** 10.3390/md20050309

**Published:** 2022-04-30

**Authors:** Concetta Maria Messina, Rosaria Arena, Simona Manuguerra, Laura La Barbera, Eleonora Curcuraci, Giuseppe Renda, Andrea Santulli

**Affiliations:** 1 Laboratorio di Biochimica Marina ed Ecotossicologia, Dipartimento di Scienze della Terra e del Mare DiSTeM, Università degli Studi di Palermo, Via G. Barlotta 4, 91100 Trapani, Italy; rosaria.arena@unipa.it (R.A.); simona.manuguerra@unipa.it (S.M.); eleonora.curcuraci@unipa.it (E.C.); giuseppe.renda02@unipa.it (G.R.); andrea.santulli@unipa.it (A.S.); 2 Istituto di Biologia Marina, Consorzio Universitario della Provincia di Trapani, Via G. Barlotta 4, 91100 Trapani, Italy; labarbera@consunitp.it

**Keywords:** side streams, ω-3 fatty acids, tuna fish oils, SAF-1 cell line, biomarkers, lipid metabolism, adipogenesis

## Abstract

The valorization of side streams from fishery and aquaculture value-chains is a valuable solution to address one of the challenges of the circular economy: turning wastes into profit. Side streams produced after filleting of sea cage fattened bluefin tuna (*Thunnus thynnus*) were analyzed for proximate composition and fatty acid profile to evaluate the possibility of producing tuna oil (TO) as a valuable source of ω-3 polyunsaturated fatty acids (PUFA) and testing its bioactivity in vitro. Ethyl esters of total fatty acids (TFA), obtained from TO, were pre-enriched by urea complexation (PUFA-Ue) and then enriched by short path distillation (SPD) up to almost 85% of the PUFA fraction (PUFA-SPe). The bioactivity of TFA, PUFA-SPe, and ethyl esters of depleted PUFA (PUFA-SPd) were tested in vitro, through analysis of lipid metabolism genes, in gilthead sea bream (*Sparus aurata)* fibroblast cell line (SAF-1) exposed to oils. TFA and PUFA-SPd upregulated transcription factors (*pparβ* and *pparγ*) and lipid metabolism-related genes (*D6D*, *fas*, *fabp*, *fatp1*, and *cd36*), indicating the promotion of adipogenesis. PUFA-SPe treated cells were similar to control. PUFA-SPe extracted from farmed bluefin tuna side streams could be utilized in fish feed formulations to prevent excessive fat deposition, contributing to improving both the sustainability of aquaculture and the quality of its products.

## 1. Introduction

Global fish production, according to FAO statistics [1], reached about 179 million tons in 2018; 156 million tons were used for human consumption, and 22 million tons were for non-food uses, mainly to produce fishmeal and fish oil.

Fish processing and its significant expansion have led to increasing amounts of side stream (about 60% of processed fish) [1,2,3]. The worldwide production of fishery wastes has many implications for environmental and economic management sustainability and the protection of marine resources [4].

The need to combine the sustainable management of marine resources with strong actions to recover the intrinsic value of side streams from fisheries and aquaculture is becoming a priority [5,6,7]. Actually, these side streams may represent unused or underutilized resources that still contain a wide number of components with high nutritional value [8,9,10]. There is a growing interest in research and industry for the sustainable use of fisheries and marine organisms and processing side streams for the extraction of high biological value molecules [4,6,7,8,11,12,13,14], especially side streams which can be used to obtain molecules of biochemical interest such as fatty acids, peptides, antioxidants, and bioactive metabolites [4,13,14,15,16,17,18], which can be utilized in nutraceutical, pharmaceutical, and animal feed fields [4,6,7,8,11,12].

The use of side streams and their valorization might lead to a reduction in product losses and food waste, generating economic development from a resource that would otherwise be discarded, ensuring the achievement of the “zero waste” goal [5,6,19,20,21].

The valorization of biological waste from fishery and aquaculture is fundamental for implementing circularity in the bioeconomy and minimizing the environmental impact [5,6,19,20,21].

Processing side streams are an optimal resource for fish oil extraction [4,6,19,22], which composition varies considerably depending on the species and fishery season [8].

Fish oils represent the main natural sources of long-chain ω-3 polyunsaturated fatty acids (PUFA), such as eicosapentaenoic acid (EPA, 20:5ω-3) and docosahexaenoic acid (DHA, 22:6ω-3), both used in aquaculture (feeds) and for direct human consumption (nutritional supplement capsules) [4,23]. Furthermore, ω-3 PUFA are of great interest to the pharmaceutical and food industries [4,24]. In fact, thanks to the numerous studies that have shown the beneficial effects on human health, there has been an exponential growth in the market of the ω-3 PUFA for human consumption [6,25,26,27,28,29,30,31,32,33].

Even research for alternative lipid sources has grown considerably in recent years. Among them, vegetable oils, alternative marine oils (krill, copepod, and amphipod oils), etc. [34] are the main lipid resources for aquatic fodder.

The increasing utilization of vegetable oils in fish feed has caused a decrease in EPA and DHA, affecting lipid metabolism, fish fillet composition, and fatty acid quality [35]. Vegetable oils are rich in α-linolenic acid (ALA, 18:3ω-3), and marine fish have a low capacity to convert ALA into EPA and DHA, which are essential for them [34,36].

Developing strategies to prevent excessive fat deposition in farmed fish could be useful for their welfare, increasing the final product quality [37], and also enhancing the sustainability of the aquaculture industry [38]. Fish oil, rich in ω-3 fatty acid, is known to regulate both the storage and secretory functions of adipose tissue [39,40]. It may also contain trans and saturated fatty acids that can have negative effects; therefore, it is needed to increase the ω-3 PUFA levels, particularly EPA and DHA, through enrichment. [41]. There are several methods to enrich ω-3 PUFA, such as chromatographic separation, fractional or molecular distillation, low-temperature crystallization urea complexation, supercritical CO_2_ extraction, and enzymatic purification [42,43,44].

Enrichment by urea complexation, using inexpensive solvents and simple equipment, is an efficient method for DHA enrichment. Furthermore, this technique has been shown to protect DHA from autoxidation [42,43,45,46].

Short-path distillation (SPD) allows the distillation of temperature-sensitive products. This process is suitable for the treatment of lipids as it is a continuous separation process working under vacuum conditions with low evaporation temperature and short sample residence time [47].

For the ω-3 PUFA enrichment, a combination of techniques, such as urea complexation followed by molecular distillation, are often used [48].

The aim of this study was to valorize the side streams obtained by the filleting of bluefin tuna (*Thunnus thynnus*) specimens BFT-F fattened in a sea cage in Castellammare del Golfo (Italy) by the production of ω-3 polyunsaturated fatty acids tuna oil (TO). BFT fattening in the Mediterranean Sea was set up as a seasonal activity intended to introduce tuna from the wild into sea cages for three months, feed them with highly energetic fish to increase the body fat percentage, and obtain a better yield in the brief fishing period [49]. In our study, BFT side streams (heads, fins, tails, and individual organs and tissues) were analyzed for proximate composition and fatty acid (FA) profile and for tuna oil (TO) extraction. Total fatty acids (TFA) extracted from the oil were enriched trough the combination of two techniques: urea complexation (PUFA-Ue), followed by short path distillation (SPD) (PUFA-SPe). The effects of TFA, PUFA-SPe, and ethyl esters depleted PUFA (PUFA-SPd), were tested in vitro in gilthead sea bream *(Sparus aurata)* fibroblast cell line (SAF-1) to investigate the effect on adipogenesis and their potential use in fish feed formulation.

## 2. Results and Discussion

### 2.1. Recovery of Processing Side Stream from Sea Cage Fattened Bluefin Tuna

Raw fish oil quality depends mainly on the raw material used for its production: the better the quality, the fattier and fresher the fish. The lipid content and the proximate composition of fish side stream changes according to the species, season, and growing conditions [50].

Results were compared with those obtained from the analysis of side streams of fresh wild bluefin tuna (BFT-W) and frozen yellowfin tuna (*T. albacares*) (YFT-W).

The obtained results (Table 1) showed a higher total lipid content in BFT-F compared to BFT-W and YFT-W (Table 1).

The high lipid content observed in the BFT-F side streams was in agreement with Šimat et al. [50] and was determined by the rearing and feeding conditions that are responsible for the increased fat deposition [49]. This observation highlights the suitability of this matrix for fish oil production [50].

Since BFT-F, due to its high lipid content, represents the most suitable matrix for fish oil extraction, the fatty acid profile was evaluated (Table 2).

Monounsaturated fatty acids (MUFA) were the most abundant class of total fatty acids (36.40 ± 1.36%); the major fatty acid was 18:1ω-9. The second most abundant class was PUFA (33.22 ± 1.72%), followed by saturated fatty acids (SFA) (30.38 ± 0.71%), and the most important SFA was palmitic acid (C16:0; 20.16 ± 0.96) (Table 2).

Regarding PUFA, bluefin tuna is considered a good source of ω-3 fatty acids, especially DHA; in fact, DHA (13.64 ± 0.56%) was higher than EPA (9.93 ± 0.36%) (Table 2). The sum of DHA and EPA reached about 24%. Similar results were observed in wild and farmed bluefin tuna muscle [51].

These results (Table 2) show that BFT-F side streams have significant EPA and DHA contents, confirming their possible utilization for the extraction of oils rich in ω-3 fatty acids [52].

### 2.2. Extraction, Yield and Quality of Tuna Oil (TO)

TO extraction was performed at 60 °C for 30 min, identified as optimal conditions by Messina et al. [6], and subsequently refined. The yield obtained was 75 ± 4.52%, higher compared to Ferdosh et al. [53] from long-tail tuna (*Thunnus tonggol*) heads. This difference depends on species, by-products used, and the extraction technique employed. De la Fuente et al. [54] reported that fish oil extracted from Atlantic salmon by-products showed different yields in relation to the by-products used (57 ± 1%, 56 ± 2%, and 77 ± 2% for spines, heads, and viscera, respectively).

In our study, high lipid content in the side streams by BFT-F (Table 1) combined with the extraction efficiency resulted in high yields.

The quality results of TO and their comparison with cod liver oil (CO) are shown in Table 3.

Among the quality parameters for oil, the peroxide value (PV) was evaluated. This parameter allows for the assessment of the rancidity of the oil [55], monitoring the formation of hydroperoxides [22,56]. The PV obtained was 2.96 meq O_2_/kg, which is comparable to the PV values obtained by Šimat et al. [22,50] in oil obtained from tuna by-products. The TO may be suitable for human consumption as it is below ≤5 meq O_2_/kg [22,57].

Other parameters related to the oxidation were evaluated, such as ρ-anisidine (ρ-AV), thiobarbituric acid reactive substances (TBARS), and total oxidation value (TOTOX) (Table 3). The observed values were lower than those obtained by Šimat et al. [22,50] on tuna by-products but were comparable to the values observed by Franklin et al. [58] in oil extracted from yellowtail fish waste by supercritical CO_2_ extraction. A lower TBARS value is related to the use of adsorbents, such as charcoal powder and Fuller’s earth, known for their capacity to adsorb primary and secondary oxidation compounds [59]. In addition, the ρ-AV, 12.93 ± 1.53, was below the limit for the acceptability of fish oil for human consumption, equal to ≤20% [60].

The low values (2.25%) of FFA in TO confirmed that the temperatures during extraction and refining did not cause significant hydrolysis, as observed by Šimat et al. [22,50]. The observed value was less than 3%, as recommended for edible oils [57,61]. It is important to obtain low FFA values since high values might lead to difficulties in the ω-3 extraction [22,56].

### 2.3. Pre-Enrichment by Urea Complexation

Total methyl esters FAs were extracted, ethyl esters (TFA) were produced by transesterification of the FAs, and preliminary fractionation by urea complexation was carried out.

Through urea complexation, a simple, quick, and efficient technique, it was possible to separate FAs according to their degree of unsaturation [62,63].

It was observed that the urea complexation treatment was efficient in extracting polyunsaturated fatty acid methyl esters and used methanol as a solvent [64]. This technique is easy to scale-up, it is environmentally sustainable, and it has been used to valorize byproducts of fish in the canning industry [64].

FA profiles are shown in Table 4.

The TO showed a predominance of MUFA and PUFA content (Table 4) greater than 50% of total FA, suggesting that oils extracted from BFT-F side streams are a rich source of unsaturated FA, as reported by Šimat et al. [50].

The variation in the percentage of PUFA and SFA in PUFA-Ue was calculated according to Equation (4) (Section 3.5). An increase equal to 86% was observed in PUFA content, while a 45% decrease was observed in SFA. In particular, an increase in DHA (22:6ω-3) was observed, with a final value equal to 22.50 ± 0.45% (Table 4).

A significant increase in PUFA through this selective enrichment was previously observed by several authors [41,60,62,63].

The use of this technique is advantageous not only for its operational simplicity but also because it is environmentally friendly, as inexpensive and renewable materials (urea and ethanol or methanol as a solvent) are used [63,65].

Polyene index (PI), which indicates PUFA damage and oxidation, showed a significant increase (*p* < 0.05) after urea pre-enrichment.

In general, a decrease in PI values in fish oil suggests the degradation of PUFA [50]; in our study, the increase in PI was related to the significant increase in PUFA, particularly DHA (Table 4).

The lipid quality indices, atherogenetic index (AI), and the thrombogenetic index (TI), which indicate the overall dietary quality of lipids and their potential effect on coronary heart disease prevention [66,67], showed an improvement in oil quality, highlighting significantly lower values due to the significant increase in PUFA and decrease in SFA and MUFA (*p* < 0.05) [66] (Table 4).

### 2.4. PUFA Enrichment by SPD

The PUFA-Ue was subjected to three cycles of SPD to enrich the PUFA content in the PUFA-SPe fraction, via the elimination of the fraction containing short-chain fatty acid ethyl esters (PUFA-SPd). Table 5 shows the differences among the PUFA-Ue, PUFA-SPe, and PUFA-SPd fatty acid profiles.

Using the SPD technique, SFA and short-chain fatty acids were distilled into the light phase [48,68]. In fact, in PUFA-SPe, a significant decrease (*p* < 0.05) of SFA (4.73 ± 0.03%) was observed compared to PUFA-Ue (17.31 ± 1.51%). In PUFA-SPd, the SFA amount was significantly increased (*p* < 0.05) compared to PUFA-Ue, reaching a value of 25.13 ± 0.20% (PUFA-SPd) (Table 5).

The SPD temperature used (160 °C), defined in previous studies [6,7], allowed us to obtain a PUFA content of 86.76 ± 0.07 (Table 5). PUFA were increased in the PUFA-SPe (from 57.53% to 86.76%), while those in the PUFA-SPd fraction were depleted (38.61%) (Table 5).

Higher temperatures (around 200 °C), might compromise PUFA, causing their degradation [69,70].

The increase in PUFA in PUFA-SPe was confirmed by the R-parameter, indicating the ratio of the sum of EPA (20:5ω-3) and DHA over the sum of 16:0 and 18:1 [69]. R increased significantly (*p* < 0.05) in PUFA-SPe (13.95 ± 0.84), while decreased in PUFA-SPd (0.73 ± 0.01) (*p* < 0.05) in respect to PUFA-Ue (Table 5).

In addition, in PUFA-SPe, a significant increase of EPA (17.59 ± 0.59%) and DHA (46.51 ± 0.34) values were observed. Furthermore, EPA Enrichment Factor (1.54 ± 0.05) DHA Enrichment Factor (1.99 ± 0.01), and PUFA Enrichment Factor (1.45 ± 0.02) showed a significant increase value (*p* < 0.05) compared to PUFA-Ue.

One of the main advantages of SPD is that it does not require chemical treatments during processing, and refined fish oils can be obtained as described by Oliveira et al. [68]. Solaesa et al. [48] demonstrated that SPD could contribute to the concentration of omega-3 PUFA obtained from an enzymatic glycerolysis of sardine oil.

The results obtained suggest that SPD is an effective separation technology that can be used to concentrate PUFA, particularly EPA and DHA, as ethyl esters from fish oil [6,48,68,71,72,73].

### 2.5. Effects of Fatty Acids Ethyl Esters on Lipid Accumulation and Related Genes, in SAF-1 Cell Lines

The development of new strategies to increase sustainable fish production and ensuring a high-quality product is among the objectives of the aquaculture sector [74]. Adjusting the dietary fatty acid composition by using different fish oils might be important to improve the flesh lipid content in fish. The mechanisms of how fatty acids affect fat deposition in fish flesh are not clearly known. In vitro systems are useful tools to identify the possible adipogenic effects of dietary lipids in fish feeds [75].

The fatty acid composition in fish tissue depends mainly on dietary fatty acids. The replacement of dietary fish oils with vegetable oils that are devoid of PUFA, alters the fatty acid composition, production value, and β-oxidation capacity [76].

**Figure 1:**
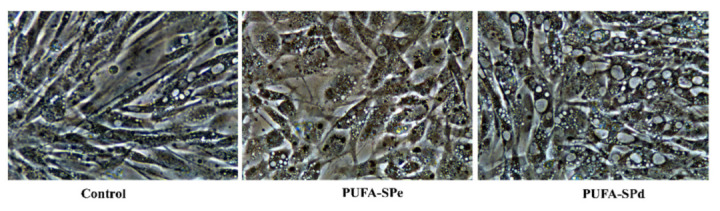
Representative phase-contrast images at 40× magnification (scale bar = 100 μm). Untreated cells (Control), SAF-1 cell treated with enriched in PUFA (PUFA-SPe), and exhausted in fatty acid ethyl esters (PUFA-SPd).

In our study on the *Sparus aurata* cell line, changes in response to the treatments were observed, starting from cellular morphology. Figure 1 shows that cells treated with PUFA-SPe showed lower lipid accumulation than those exposed to PUFA-SPd. PUFA-SPd treated cells were more rounded with an enlarged cytoplasm, losing the fibroblastic shape and a significant lipid accumulation, suggesting that PUFA-SPd might stimulate fat uptake and fat cytoplasm accumulation. These findings are in agreement with the anti-adipogenic effects of PUFA during pre-adipocyte differentiation in cobia (*Rachycentron canadum*) [75], Atlantic salmon (*Salmo salar*) [77], and rainbow trout (*Oncorhynchus mykiss*) [78].

Adipogenesis is a consequence of normal cell turnover because of the need to store energy [37], and high concentrations of fatty acids play an essential role in adipocyte differentiation [79]. Very little is known about the regulation and development of adipose tissue in fish. ω-3 PUFAs (EPA and DHA) are able to inhibit adipocyte differentiation and decrease lipid accumulation through the down-regulation of certain transcriptional factors or lipolytic genes [80].

In this study, lipid metabolism-related genes were analyzed to investigate how the predominant fatty acids contained in TFA, PUFA-SPe, and PUFA-SPd, affect molecular pathways associated with lipid accumulation in vitro.

**Figure 2:**
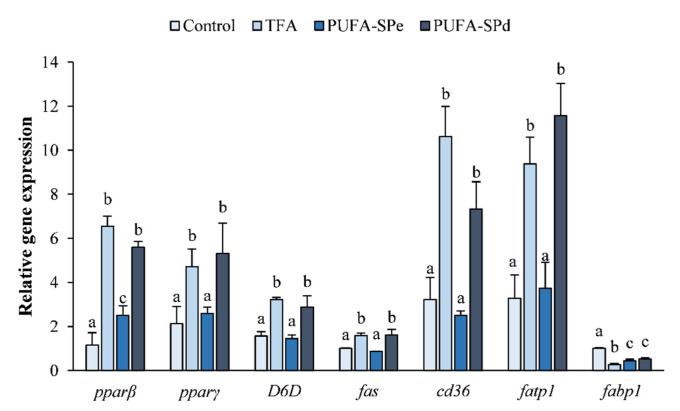
Relative expression of transcription factors (*pparβ* and *pparγ*), lipid metabolism-related genes (*D6D and fas*) and fatty acid transporters (*cd36*, *fatp1*, *fabp1*) in SAF-1 cell line treated or not (Control) with total FA ethyl esters (TFA), enriched in PUFA (PUFA-SPe), and exhausted in fatty acid ethyl esters (PUFA-SPd). Date are shown as mean ± SEM (*n* = 3). Different letters indicate significant differences among treatments (*p* < 0.05). Peroxisome proliferator-activated receptor β (*pparβ*), peroxisome proliferator-activated receptor γ (*pparγ*), Delta 6 desaturase (*D6D*), fatty acid synthase (*fas*), fat translocase/cluster of differentiation (*cd36*), fatty acid transporter protein 1 (*fatp1*) and fatty acid binding protein (*fabp1*).

Peroxisome proliferator-activated receptors (PPAR) are nuclear receptor proteins that function as fatty acid-activated transcription factors with a key regulatory role in lipid metabolism [81]. *pparβ* and *pparγ* are target genes involved in adipocyte differentiation and in lipid storage [81,82]. In our study, *pparβ* and *pparγ* genes were significantly upregulated (*p* < 0.05) in SAF-1 cells treated with TFA and PUFA-SPd, compared to no-treated cells (control) (Figure 2). *pparβ* and *pparγ* mRNA levels in cells treated with PUFA-SPe were similar to the control (Figure 2). Li et al. [83] reported that dietary fish oil decreased *pparγ* gene expression in intraperitoneal fat of *Ctenopharyngodon idellus,* suggesting that ω-3 PUFA could inhibit lipid accumulation by affecting the expression of lipid-metabolism-related genes.

These results are in accordance with the anti-adipogenic effect of EPA and DHA observed in several in vitro studies: *pparγ* results showed that reduced preadipocyte 3T3-L1 induced differentiation in the presence of EPA and DHA alone or combined, indicating that both FA attenuated adipogenesis [40,84]. Even in fish adipocytes cells, PUFA suppressed the expression of *pparβ*, and fatty acid transport protein (*fatp1*) during differentiation [76]. Although, other authors have demonstrated a pro-adipogenic effect of these two fatty acids in similar mammalian cell line models [39,85,86]. Nevertheless, the effect of fatty acids in the modulation of adipogenesis and lipid accumulation in fish is very complex and not completely understood yet.

The lipid composition of an organism is not only dependent on the dietary lipids ingested but also on the capacity of the species to transform these lipids through desaturation and elongation pathways [87]. Marine fish have a lower capacity for the bioconversion of 18C fatty acid precursors into PUFA, hence requiring the preformed PUFA in their diet [87]. Delta 6 desaturase (D6D) is a key enzyme in the biosynthesis of ω-3 PUFA from precursors [34,88]. Seiliez et al. [89] reported that *D6D* gene expression in sea bream liver was high in fish fed with a PUFA-free diet (containing vegetable oil as a lipid source).

In the current study, the *D6D* gene expression was significantly upregulated (*p* < 0.05) in cells treated with TFA and PUFA-SPd compared to the control (Figure 2), whereas in the PUFA-SPe treatment, it was similar to the control (Figure 2). These results suggest that *D6D* expression could be inhibited by high levels of dietary DHA content, as reported in rainbow trout [90], and upregulated by a vegetable oil replacement diet in Meagre *Argyrosomus regius* [91].

In agreement with these studies, an increase of *D6D* was recorded in gilthead seabream fed with vegetable oil [34] and Atlantic salmon fed with diets containing linseed oil compared to fish oil [87], suggesting that the high PUFA levels present in fish oil might suppress *D6D* activity, decreasing the ability of marine fish to desaturate 18C fatty acids precursors.

The fatty acid synthase (*fas*) gene is a marker of de novo lipogenesis involved in the conversion of acetyl-CoA and malonyl-CoA to palmitate [92]. Cells incubated with TFA and PUFA-SPd were significantly upregulated (*p* < 0.05) compared to the control (Figure 2). Whereas *fas* mRNA levels in SAF-1 cells treated with PUFA-SPe were similar to the control (Figure 2), this was perhaps due to direct inhibition of de novo synthesis caused by the addition of PUFA into the culture medium [93]. These results suggest that the addition of PUFA to the diet could inhibit the lipogenic pathway, restraining lipid synthesis [78,94].

Among genes related to uptake and transport of fatty acid, we considered fat translocase/cluster of differentiation (*cd36*), fatty acid transporter protein 1 (*fatp1*), and fatty acid-binding protein 1 (*fabp1*)*. Cd36* is an integral membrane glycoprotein that transports fatty acids into adipocytes [40]. *Fatp1* are membrane-bound fatty acid uptake proteins that have an important role in responding to changes in metabolic activity [81]. Finally, *fabp1* is a protein that transports fatty acids through the cytoplasm [81,95].

In our experiment, both *cd36* and *fatp1* resulted in significantly upregulated TFA and PUFA-SPd treated cells, with respect to the control (Figure 2), highlighting the important role in the uptake of fatty acids from the environment [93]. Whereas the PUFA-SPe treated cells were similar to the control (Figure 2). Our results are in accordance with Martins et al. [40], reporting that EPA and DHA treatment, alone or combined, downregulated *cd36* mRNA levels in 3T3-L1 cells, reducing fatty acid transport into adipocytes.

The gene *fabp1* was downregulated in all treatments compared to the control (Figure 2). These observations might indicate that ω-PUFA reduces fatty acid uptake and transport activity [93], affecting the catabolism of fatty acids [81].

Our in vitro observations confirm, as previously reported [6], that dietary PUFA have several beneficial properties for farmed fish, affecting lipid metabolism, with a possible effect on fat deposition in fish fillets. Therefore, regulating the composition of fatty acids in the diet could improve the lipid deposition in various tissues and, consequently, the lipid profile of the edible parts of fish, contributing to ameliorating the acceptance of consumers.

## 3. Materials and Methods

### 3.1. Sampling

By-products of BFT-F were sampled during harvesting and filleting on board a fishing boat, together with the by-products of BFT-W and YFT-W, collected from a fishery.

For each lot, twelve samples were done (*n* = 12).

BFT-F, BFT-W, and YFT-W were placed in cold containers and transported to the laboratory (less than one hour), and stored at −80 °C until analyses.

### 3.2. Proximate Composition and FA Profile

Proximate composition and FA profile were analyzed from the by-products of BFT-F, BFT-W, and YFT-W, homogenated on ice by Ultraturrax T25 (IKA, Labortechnik, Staufen, Germany). Each homogenate was used to assess the moisture content by the AOAC method [96] and the crude protein content was found by the Kjeldahl method, multiplying the measured total nitrogen by the conversion factor 6.25 [97]. The total lipids were determined according to Folch et al. [98] and the FA methyl esters were obtained according to Lepage and Roy [99], and their profile was obtained by gas chromatography, as described in Messina et al. [14].

The fatty acid profile was utilized to evaluate the following indices:

- the polyene index (PI) (Equation (1)), used as a measure of PUFA damage [100]:
  PI = ((C20:5ω-3 + C22:6ω-3)/C16:0)(1)
  where C20:5ω-3 represents EPA, C22:6ω-3 DHA and C16:0 palmitic acid.

- the atherogenicity index (AI) (Equation (2)), is a nutritional quality index for assessing the risk of platelet aggregation [101]:
  AI = (C12:0 + 4 × C14:0 + C16:0)/(MUFA + PUFA)(2)
  where C12:0 represents lauric acid, C14:0 miristyc acid, C16:0 palmitic acid, MUFA monounsaturated fatty acid and PUFA polyunsaturated fatty acid.

- the thrombogenicity index (TI) (Equation (3)) [101], is a nutritional quality index representing the potential to form clots in the blood vessels
  TI = [(C14:0 + C16:0 + C18:0)/(0.5 × MUFA + 0.5 × Totω-6 + 3 × Totω-3 + (Totω-3/Totω-6))](3)
  where C14:0 represents miristyc acid, C16:0 palmitic acid, C18:0 stearic acid, MUFA is monounsaturated fatty acid, Totω-6 the total amount of omega 6 fatty acid and Totω-3 the total amount of omega 3 fatty acids.

### 3.3. Extraction of Crude Oil

TO was extracted from 10 kg BFT-F side stream batches by wet extraction [6,102]. Preheated distilled water was added to the ground BFT-F side stream at a 1:2 *w*/*v* ratio, and the mixture was incubated in a 50 L steel reactor equipped with an internal heating coil. Extraction was performed under constant agitation at 60 °C for 30 min, with optimal yields condition reported by Messina et al. [6]. Extraction mixtures were filtered on a 125 µm mesh sieve to remove the coarse particulates. The filtrate liquid phase was centrifuged at a centrifugal force of 40,000× *g* by a continuous tubular centrifuge (CEPA, Carl Padberg, Zentrifugenbau GmbH, Lahr/Schwarzwald, Germany) equipped with a separating cylinder (type TR).

The extraction mixture was fed at the bottom of the cylinder by a Masterflex L/S peristaltic pump equipped with tubing L/S 18 (Cole-Parmer s.r.l., Cernusco sul Naviglio, MI, Italy), with a throughput of 0.03 L·min^−1^. This configuration allowed us to separate contemporary and continuous solids (retained in the cylinder), a heavy liquid phase (containing protein and cellular end tissue debris), and a light liquid phase (containing TO) that exited the cylinder in two separate fluxes. TO samples were refined as described by Messina et al. [6] and stored at −20 °C in 2.5 L dark bottles under nitrogen.

### 3.4. Assessment of Oil Quality

The quality of TO was evaluated by monitoring PV, TBARS, ρ-AV, TOTOX, and free fatty acid (FFA)% as described by Messina et al. [6]. Commercial cod liver oil was used as the control oil (CO) (Pearson, Italy).

### 3.5. Urea PUFA Enrichment

The batches of extracted tuna oil (2.5 L) were trans-esterified to obtain TFA without the use of any solvent other than ethanol, as reported by Vazquez and Akoh [103]. During transesterification, all liquid phase separations were carried out by a continuous tubular centrifuge equipped with a separating cylinder, as described above by Messina et al. [6].

TFA were mixed with ethanol and urea, a simple method for obtaining concentrated PUFA [104]. SFA and MUFA bind easily to urea and crystallize at low temperatures. Crystals were removed by centrifugation and subsequently recovered with n-hexane to determine saturated residual fraction. PUFA-Ue supernatant was recovered with n-hexane and evaporated to obtain a concentrated sample. The PUFA-Ue fractions obtained after treatment with urea were diluted in c-hexane to final a 1% concentration for gas-chromatographic analysis.

The variation in the percentages of SFA, MUFA, and PUFA were calculated by Equation (4):

% FA variation = (∑%FA-Ue − ∑%FA-TFA)/∑%FA-TFA × 100(4)

where ∑%FA-Ue is the percentage of FAs after urea complexation. ∑%FA-TFA is the percentage of FAs in the TFA [60].

### 3.6. Shorth Path Distillation PUFA Enrichment

Batches of 2 L of PUFA-Ue were distilled by SPD using the VLK 70-4 molecular distillation unit (VTA Gmbh, Niederwinkling, Germany) with an evaporating surface of 4.8 dm^2^.

Before PUFA enrichment, to remove impurities and any solvent traces, PUFA-Ue underwent a degassing step.

PUFA-Ue samples, preheated to 40 °C, were loaded into the feed vessel (at 40 °C) by a peristaltic pump. Distillation trials were run utilizing the following operating conditions: feeding vessel at 40 °C, condenser at 25 °C; evaporator at 160 °C (120 °C for degassing); feeding rate of 300 mL/h (500 mL/h for degassing); roller speed of 400 rpm; and vacuum of < 0.01 mbar (5 mbar for degassing).

A second enrichment test was performed by processing the ethyl esters at the evaporation temperature (160 °C), at which the highest PUFA concentration was obtained, and three cycles were repeated at the same temperature on the same enriched fraction to obtain a further concentration.

PUFA-SPe (heavy phase) and PUFA-SPd (distilled phase) were collected, and yields were determined gravimetrically.

In order to evaluate the enrichment process for every 1.0 L PUFA-Ue feed and at the end of distillation, aliquots of the two separated phases were diluted at 1% in c-hexane to analyze FA profiles by GC.

On the basis of the FA profile, the following indices were calculated:

- EPA and DHA%.
- FA ratio (R) (Equation (5)) [69]:
  R = (C20:5ω-3 + C22:6ω-3)/(16:0 + 18:1ω9)(5)
  where C20:5ω-3 represents EPA, C22:6ω-3 DHA, 16:0 palmitic acid and 18:1ω9 oleic acid.

- Enrichment factor for EPA, DHA, and PUFA.
- Ratio of total PUFA to total SFA (PUFA/SFA).

### 3.7. SAF-1 Cell Culture

The SAF-1 cell line (ECACC No. 00122301) was cultured at 25 °C in Leibovitz L-15 medium supplemented with 2% l-glutamine, 100 U mL^−1^ penicillin, 100 μg mL^−1^ streptomycin and 15% foetal bovine serum (all reagents from Sigma-Aldrich, Saint Louis, MO, USA). Cells were seeded in 12-well plates (Nunc, Germany) (500,000 cells/well) and incubated for 24 h.

After 24 h, the cells were treated with TFA, PUFA-SPe, and PUFA-SPd dissolved in ethanol at a concentration of 5 μg/mL in the medium, with a final solvent concentration of 0.1% (*v/v*), and left to incubate for 48 h. As attested by routine internal procedures [6], ethanol did not exert any detrimental effects when used as a vehicle.

Then, the medium was removed and cells were washed using PBS, and 1 mL of PUREzol (Bio-Rad, Hercules, CA, USA) was added.

### 3.8. Image Acquisition

Cells were observed daily using an inverted microscope Nikon Eclipse Ti-S (Nikon Instrument Inc., Melville, NY, USA), and the images were obtained with a Nikon DS-L3 digital camera (Nikon Corporation, Tokyo, Japan) and the DS-L3 Digital Camera Controller acquisition software. The images represent the SAF-1 cells observed with phase-contrast microscopy at 40× magnification.

### 3.9. RNA isolation and Rt-qPCR

The total RNA was isolated from SAF-1 cells using the RNA extraction kit (Aurum Total RNA Fatty and Fibrous Tissue Kit (Bio-Rad, Hercules, CA, USA). Then, 1 μg of the total RNA was converted into cDNA using the 5X iScript Reaction Mix Kit (Bio-Rad, Hercules, CA, USA). RT-qPCR was performed in a 20 μL reaction system using the 1X IQ SYBR Green Supermix (Bio-Rad, Hercules, CA, USA). The relative mRNA level of a target gene was quantified using the comparative cycle threshold (2^−ΔΔCT^) method [105]. The *18s* of the endogenous reference and the relative quantification of (*pparβ, pparγ, D6D, fas, cd36, fatp1*, and *fabp1*) gene expression was evaluated after normalization with the reference genes. Data processing and statistical analyses were performed using the CFX Manager Software (Bio-Rad, Hercules, CA, USA). The primers used are shown in Table 6.

### 3.10. Statistical Analysis

Results are reported as mean ± standard deviation. A comparison of the two treatments was conducted using the Student’s *t*-test. Statistical differences among different treatments were evaluated for each parameter with analysis of variance (ANOVA). Student-Newman-Keuls post-hoc tests were performed in order to make multiple comparisons between experimental groups. The degree of heterogeneity was measured by the Cochran test [43]. Differences were considered statistically significant when *p* < 0.05. Data were processed by Statistica (version 8.0, Statsoft, Inc., Tulsa, OK, USA).

## 4. Conclusions

The utilization of farmed bluefin tuna side streams could be linked to the fish processing sector, generating development and efficiency from a resource that would otherwise be discarded. Reducing food losses and waste, as well as valorizing food waste, is fundamental to achieving zero waste.

PUFA-SPe extracted from farmed bluefin tuna side streams could be utilized in fish feed formulations to prevent excessive fat deposition, contributing to improving both aquaculture sustainability and the quality of its products.

In addition, the ω-3 PUFA side stream of farmed bluefin tuna could contain other compounds such as collagen, minerals, and proteins with a high biological value that could be obtained after the extraction of the oil for their application in animal feed, cosmeceutical, and nutraceutical fields.

## Figures and Tables

**Table 1:** Proximate composition (mean ± SD) (g/100 g) of total side stream minced from wild (BFT-W), farmed (BFT-F < 60 kg; BFT-F > 200 kg) bluefin tuna, and wild yellowfin tuna (YFT-W).

	Moisture	Ash	Total Lipid	Protein
BFT-W	62.1 ± 2.0 ^c^	4.8 ± 0.4 ^a^	19.6 ± 1.5 ^b^	13.3 ± 3.4 ^a^
BFT-F > 200 kg	46.8 ± 1.8 ^a^	5.52 ± 2.1 ^a^	32.1 ± 2.1 ^c^	14.2 ± 1.8 ^a^
BFT-F < 60 kg	57.1 ± 1.1 ^b^	4.25 ± 0.5 ^a^	22.5 ± 1.2 ^b^	19.2 ± 2.4 ^b^
YFT-W	61.0 ± 1.5 ^c^	6.4 ± 1.1 ^b^	10.8 ± 0.8 ^a^	20.5 ± 3.2 ^c^

Means in the same column with different superscript letters are significantly different (*p* < 0.05). The data are reported as mean ± standard deviation, *n* = 12.

**Table 2:** FA profile (relative percentage with respect to total FAs) of total pooled side streams from BFT-F > 200 kg.

FA	BFT-F
14:0	5.79 ± 0.18
16:0	20.16 ± 0.96
16:1ω-7	6.84 ± 0.45
16:2ω-4	1.03 ± 0.02
16:3ω-4	0.56 ± 0.19
18:0	4.43 ± 0.13
18:1ω-9	15.71 ± 0.61
18:1ω-7	6.78 ± 0.27
18:2ω-6	1.22 ± 0.03
18:3ω-3	6.03 ± 0.59
20:1ω-9	2.77 ± 0.09
20:4ω-6	0.54 ± 0.01
20:4ω-3	0.29 ± 0.01
20:5ω-3 (EPA)	9.93 ± 0.36
22:1ω-11	4.30 ± 0.57
22:6ω-3 (DHA)	13.64 ± 0.56
SFA	30.38 ± 0.71
MUFA	36.40 ± 1.36
PUFA	33.22 ± 1.72
Totω-3	29.89 ± 1.49
Totω-6	1.75 ± 0.03
EPA + DHA	23.57 ± 0.92
PI	1.17 ± 0.09
AI	0.62 ± 0.01
AT	0.24 ± 0.01

The data are reported as mean ± standard deviation, *n* = 12. SFA Saturated fatty acids; MUFA Monounsaturated fatty acids; PUFA Polyunsaturated fatty acids; PI polyene index; AI atherogenicity index; TI thrombogenicity index.

**Table 3:** TO quality determined by: Peroxide Value: PV; ρ-Anisidine: ρ-AV; Thiobarbituric acid reactive substances: TBARS; Total Oxidation Value: TOTOX; Acid value and Free Fatty Acid percentage: FFA%. Commercial Cod liver oil (CO) was used as control.

	TO	CO
PV (meq O_2_/kg)	2.96 ± 0.55 ^b^	2.10 ± 0.53 ^a^
ρ-AV	12.93 ± 1.53 ^b^	4.96 ± 0.89 ^a^
TBARS (MDA µg/g)	11.81 ± 2.45 ^b^	5.51 ± 0.81 ^a^
TOTOX	20.80 ± 0.88 ^b^	10.83 ± 0.24 ^a^
Acid value (FFA%)	2.25 ± 0.32 ^b^	0.50 ± 0.04 ^a^

Different superscript letters in the same raw indicate significant differences (*p* < 0.05). The data are reported as mean ± standard deviation (*n* = 12).

**Table 4:** FA profile (relative percentage with respect to total FAs) of total methyl esters in Tuna Oil (TO), total FA ethyl esters (TFA), and samples pre enriched by urea complexation (PUFA-Ue).

FA	TO	TFA	PUFA-Ue
14:0	6.82 ± 0.11 ^b^	6.99 ± 0.10 ^b^	4.37 ± 0.18 ^a^
16:0	21.15 ± 0.31 ^c^	19.38 ± 0.22 ^b^	9.21 ± 0.23 ^a^
16:1ω-7	7.27 ± 0.09 ^c^	6.24 ± 0.09 ^b^	4.09 ± 0.06 ^a^
16:2ω-4	1.53 ± 0.19 ^b^	1.34 ± 0.11 ^b^	0.27 ± 0.03 ^a^
16:3ω-4	0.84 ± 0.13 ^b^	0.79 ± 0.03 ^b^	0.41 ± 0.08 ^a^
18:0	3.75 ± 0.22 ^b^	2.85 ± 0.12 ^a^	3.73 ± 0.05 ^b^
18:1ω-9	15.42 ± 0.4 ^c^	13.80 ± 0.3 ^b^	8.52 ± 0.35 ^a^
18:1ω-7	6.66 ± 0.13 ^b^	6.96 ± 0.31 ^b^	3.67 ± 0.28 ^a^
18:2ω-6	1.21 ± 0.02 ^a^	3.25 ± 0.08 ^b^	5.29 ± 0.09 ^c^
18:3ω-6	0.44 ± 0.29 ^a^	0.99 ± 0.25 ^a^	2.50 ± 0.12 ^b^
18:3ω-3	0.92 ± 0.09 ^a^	0.89 ± 0.15 ^a^	8.47 ± 0.1 ^b^
20:1ω-9	3.00 ± 0.42 ^a^	3.04 ± 0.18 ^a^	3.66 ± 0.34 ^a^
20:4ω-6	0.59 ± 0.04 ^a^	0.34 ± 0.21 ^a^	2.38 ± 0.22 ^b^
20:4ω-3	0.44 ± 0.08 ^a^	0.20 ± 0.13 ^a^	1.78 ± 0.07 ^b^
20:5ω-3	9.73 ± 0.35 ^a^	10.26 ± 0.05 ^a^	10.96 ± 0.03 ^b^
22:1ω-11	4.84 ± 0.35 ^a^	5.02 ± 0.32 ^a^	5.06 ± 0.18 ^a^
22:1ω-9	0.16 ± 0.05 ^a^	0.14 ± 0.03 ^a^	0.16 ± 0.04 ^a^
22:4ω-6	0.41 ± 0.10 ^a^	1.37 ± 0.29 ^b^	1.53 ± 0.09 ^c^
22:5ω-3	1.21 ± 0.05 ^a^	1.25 ± 0.09 ^a^	1.43 ± 0.012 ^b^
22:6ω-3	13.60 ± 0.08 ^a^	14.90 ± 0.16 ^b^	22.50 ± 0.45 ^c^
SFA	31.72 ± 0.90 ^a^	29.23 ± 1.21 ^a^	17.31 ± 1.51 ^b^
MUFA	37.36 ± 1.88 ^c^	35.19 ± 1.18 ^b^	25.16 ± 1.18 ^a^
PUFA	30.92 ± 1.08 ^a^	35.58 ± 1.19 ^b^	57.53 ± 1.35 ^c^
Totω-3	25.90 ± 0.65 ^a^	27.50 ± 0.95 ^b^	45.14 ± 1.75 ^c^
Totω-6	2.20 ± 0.38 ^a^	4.96 ± 0.34 ^b^	9.20 ± 0.86 ^c^
EPA + DHA	23.33 ± 0.98 ^a^	25.15 ± 0.75 ^b^	33.46 ± 1.02 ^c^
PI	1.10 ± 0.02 ^a^	1.30 ± 0.03 ^b^	3.63 ± 0.25 ^c^
AI	0.71 ± 0.09 ^b^	0.67 ± 0.09 ^b^	0.32 ± 0.02 ^a^
TI	0.29 ± 0.04 ^b^	0.27 ± 0.02 ^b^	0.11 ± 0.01 ^a^

Different superscript letters in the same row indicate significant differences (*p* < 0.05). The data are reported as mean ± standard deviation, *n* = 12.

**Table 5:** FA profile (relative percentage with respect to total FAs) of the oils, pre enriched by urea (PUFA-Ue), enriched in PUFA (PUFA-SPe), and exhausted in fatty acid ethyl esters (PUFA-SPe), obtained by short path distillation (SPD) at 160 °C.

FA	PUFA-Ue	PUFA-SPe	PUFA-SPd
14:0	4.37 ± 0.18 ^b^	0.86 ± 0.03 ^a^	6.39 ± 0.49 ^c^
16:0	9.21 ± 0.23 ^b^	2.23 ± 0.06 ^a^	14.38 ± 0.53 ^c^
16:1ω-7	4.09 ± 0.06 ^b^	0.99 ± 0.01 ^a^	5.99 ± 0.12 ^c^
16:2ω-4	0.27 ± 0.03 ^b^	0.22 ± 0.01 ^b^	0.07 ± 0.03 ^a^
16:3ω-4	0.41 ± 0.08 ^b^	0.26 ± 0.00 ^a^	0.80 ± 0.12 ^c^
18:0	3.73 ± 0.05 ^b^	1.64 ± 0.06 ^a^	4.37 ± 0.24 ^c^
18:1ω-9	8.52 ± 0.35 ^b^	2.39 ± 0.19 ^a^	14.58 ± 0.11 ^c^
18:1ω-7	3.67 ± 0.28 ^b^	0.95 ± 0.03 ^a^	4.52 ± 0.14 ^c^
18:2ω-6	5.29 ± 0.09 ^b^	3.14 ± 0.06 ^a^	5.29 ± 0.67 ^a^
18:3ω-6	2.50 ± 0.12 ^b^	1.48 ± 0.16 ^a^	2.86 ± 0.23 ^b^
18:3ω-3	8.47 ± 0.10 ^b^	8.33 ± 0.10 ^b^	4.99 ± 0.28 ^a^
20:1ω-9	3.66 ± 0.34 ^b^	1.29 ± 0.06 ^a^	5.70 ± 0.27 ^a^
20:4ω-6	2.38 ± 0.22 ^b^	2.63 ± 0.13 ^b^	0.48 ± 0.08 ^a^
20:4ω-3	1.78 ± 0.07 ^b^	1.77 ± 0.00 ^b^	0.85 ± 0.06 ^a^
20:5ω-3	10.96 ± 0.03 ^b^	17.59 ± 0.59 ^c^	4.85 ± 0.45 ^a^
22:1ω-11	5.06 ± 0.18 ^b^	2.10 ± 0.06 ^a^	5.20 ± 0.17 ^b^
22:1ω-9	0.16 ± 0.04 ^a^	0.79 ± 0.00 ^b^	0.26 ± 0.05 ^a^
22:4ω-6	1.53 ± 0.09 ^b^	1.86 ± 0.03 ^c^	0.68 ± 0.13 ^a^
22:5ω-3	1.43 ± 0.12 ^a^	2.97 ± 0.20 ^b^	1.49 ± 0.01 ^a^
22:6ω-3	22.50 ± 0.45 ^b^	46.51 ± 0.34 ^c^	16.25 ± 0.14 ^a^
SFA	17.31 ± 1.51 ^b^	4.73 ± 0.03 ^a^	25.13 ± 0.20 ^c^
MUFA	25.16 ± 1.18 ^b^	8.51 ± 0.10 ^a^	36.26 ± 0.42 ^c^
PUFA	57.53 ± 1.35 ^b^	86.76 ± 0.07 ^c^	38.61 ± 0.22 ^a^
Totω-3	45.14 ± 1.75 ^b^	77.17 ± 0.24 ^c^	28.44 ± 0.52 ^a^
Totω-6	9.20 ± 0.86 ^c^	7.63 ± 0.20 ^b^	6.44 ± 0.87 ^a^
EPA + DHA	33.46 ± 1.02 ^b^	64.10 ± 0.25 ^c^	21.09 ± 0.31 ^a^
PI	3.63 ± 0.25 ^b^	28.81 ± 0.95 ^c^	1.47 ± 0.03 ^a^
IA	0.32 ± 0.02 ^b^	0.06 ± 0.00 ^a^	0.53 ± 0.02 ^c^
IT	0.11 ± 0.01 ^b^	0.02 ± 0.00 ^a^	0.23 ± 0.01 ^c^
R	1.89 ± 0.23 ^b^	13.95 ± 0.83 ^c^	0.73 ± 0.01 ^a^
EPA Enrichment Factor	1.00 ± 0.07 ^b^	1.54 ± 0.05 ^c^	0.90 ± 0.08 ^a^
DHA Enrichment Factor	1.00 ± 0.07 ^b^	1.99 ± 0.01 ^c^	0.69 ± 0.01 ^a^
PUFA Enrichment Factor	1.00 ± 0.07 ^b^	1.45 ± 0.02 ^c^	0.70 ± 0.01 ^a^
PUFA/SFA	3.32 ± 0.25 ^b^	18.35 ± 0.10 ^c^	1.54 ± 0.01 ^a^

Different superscript letters in the same row indicate significant differences *p* < 0.05). The data are reported as mean ± standard deviation, *n* = 12.

**Table 6:** *S. aurata* primer sequences used for real-time PCR.

Gene	F/R Primer Sequence (5′–3′)
*pparβ*	Fw CAGGTGACTTTGCTGAAGTA
*pparγ*	Rv TAAAGGGCTTTCTTAAGCTG
	Fw CTCAAGAGTCTCAGGAAACC
*D6D*	Rv GATAATGACAGCCAGAAACA
	Fw TACCTTCGCTACCTGTGCTG
*fas*	Rv TTGCAGGTGGTTGTAACTG
	Fw TGCCATTGCCATAGCACTCA
*cd36*	Rv ACCTTTGCCCTTTGTGTGGA
	Fw GTCGTGGCTCAAGTCTTCCA
*fatp1*	Rv TTTCCCGTGGCCTGTATTCC
	Fw CAACAGAGGTGGAGGGCATT
*fabp1*	Rv GGGGAGATACGCAGGAACAC
	Fw CATGAAGGCGATTGGTCTCC
	Rv GTCTCCAAGTCTGCCTCCTT
*18s*	Fw TGTGCCGCTAGAGGTGAAATT
	Rv GCAAATGCTTTCGCTTTCG

## Data Availability

Data contained within the article.
